# Medical care of asylum seekers: a descriptive study of the appropriateness of nurse practitioners' care compared to traditional physician-based care in a gatekeeping system

**DOI:** 10.1186/1471-2458-7-310

**Published:** 2007-10-31

**Authors:** Patrick Bodenmann, Fabrice Althaus, Bernard Burnand, Paul Vaucher, Alain Pécoud, Blaise Genton

**Affiliations:** 1 Medical Outpatient Clinic, Department of Ambulatory Care and Community Medicine, University of Lausanne, Rue du Bugnon 44, 1011 Lausanne, Switzerland; 2 Institute of Social and Preventive Medicine, University of Lausanne, Rue du Bugnon 17, 1011 Lausanne, Switzerland

## Abstract

**Background:**

Medical care for asylum seekers is a complex and critical issue worldwide. It is influenced by social, political, and economic pressures, as well as premigration conditions, the process of migration, and postmigration conditions in the host country. Increasing needs and healthcare costs have led public health authorities to put nurse practitioners in charge of the management of a gatekeeping system for asylum seekers. The quality of this system has never been evaluated. We assessed the competencies of nurses and physicians in identifying the medical needs of asylum seekers and providing them with appropriate treatment that reflects good clinical practice.

**Methods:**

This cross-sectional descriptive study evaluated the appropriateness of care provided to asylum seekers by trained nurse practitioners in nursing healthcare centers and by physicians in private practices, an academic medical outpatient clinic, and the emergency unit of the university hospital in Lausanne, Switzerland. From 1687 asylum seeking patients who had consulted each setting between June and December 2003, 450 were randomly selected to participate. A panel of experts reviewed their medical records and assessed the appropriateness of medical care received according to three parameters: 1) use of appropriate procedures to identify medical needs (medical history, clinical examination, complementary investigations, and referral), 2) provision of access to treatment meeting medical needs, and 3) absence of unnecessary medical procedures.

**Results:**

In the nurse practitioner group, the procedures used to identify medical needs were less often appropriate (79% of reports vs. 92.4% of reports; p < 0.001). Nevertheless, access to treatment was judged satisfactory and was similar (p = 0.264) between nurse practitioners and physicians (99% and 97.6% of patients, respectively, received adequate care). Excessive care was observed in only 2 physician reports (0.8%) and 3 nurse reports (1.5%) (p = 0.481).

**Conclusion:**

Although the nursing gatekeeping system provides appropriate treatment to asylum seekers, it might be improved with further training in recording medical history and performing targeted clinical examination.

## Background

The medical care of asylum seekers (according to the International Organization for Migration, persons who "have crossed an international border and have not yet received a decision on their claim for refugee status") is a critical issue worldwide. Each nation tries to respond in an optimal way, considering social and political pressures as well as financial resources. But no system has proven entirely satisfactory. Furthermore, healthcare providers for asylum seekers need to consider the premigration conditions, the process of migration, which is frequently forced and traumatic [1-5], and the postmigration conditions in the host country. Prevention of health problems and follow-up for asylum seekers is often far from optimal [6-10], and use of healthcare services also depends on ethnicity and medical insurance coverage [11-13]).

As with other vulnerable populations, the identification of healthcare needs and the planning of appropriate care seem to be a priority [14]. Healthcare providers serve a gatekeeping function, increasing coordination and preventive care and reducing inappropriate or duplicate care. This is accomplished by preventing overlap in the functions of healthcare providers caring for the same patient and making appropriate referrals to other care providers [15].

Following the Dutch example [16], in Western Switzerland nurses have undertaken the management of primary care for asylum seekers in a gatekeeping system [17,18]. The shift of responsibility from physicians to nurse practitioners was mainly due to the increasing and complex healthcare needs of asylum seekers, who, as frequently traumatized individuals, present multiple sociomedical demands, have increasing demands to find them medical opportunities to stay in the host country; have to face; the decreasing number of physicians and increased pressure to contain costs. In other settings, the replacement of physicians with trained nurses (nurse practitioners) does not seem to reduce the quality of primary care in terms of health outcomes, the process of care, resource utilization, or cost [19,20]. Studies have shown that bypassing the physician does not alter the efficiency of "same day" primary care [21] or community care [22]. Moreover, primary care nurses provide high quality care, particularly in difficult sociocultural contexts [23,24].

**Figure 1:**
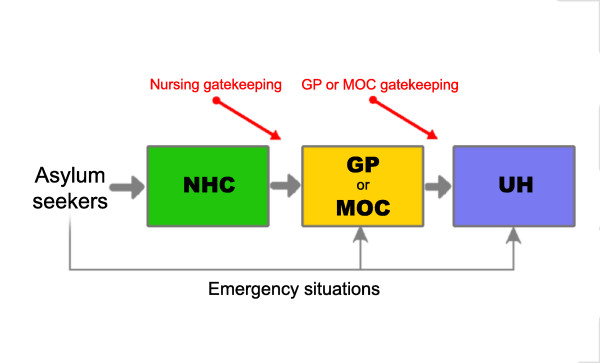
**Double gatekeeping healthcare system for asylum seekers in Western-Switzerland**. NHC: Nurse Healthcare Centre. GP: General Practitioners. MOC: Medical Outpatient Clinic. UH: University Hospital emergency ward department.

While nurse practitioners have been working for approximately 30 years in the U. S. and 15 years in the UK, this kind of service is still in its infancy in Switzerland. For asylum seekers in Western Switzerland, access to care generally requires a preliminary visit to a center managed by nurse practitioners who serve a gatekeeping function. General practitioners only intervene when asked to do so by nurse practitioners. However, emergency situations can be handled directly by physicians, medical outpatient clinics, or emergency centers (Figure 1). The quality of care for asylum seekers in this type of system is less clear. To our knowledge; no study has analyzed the appropriateness of medical care for asylum seekers using a nurse practitioner gatekeeping process. The aim of this study is to compare the appropriateness of asylum seekers' reported medical procedures whether they have been taken care of by nurse practitioners or by physicians.

## Methods

In this cross-sectional observational study, experts consulted the medical records of asylum seeking patients who had visited nurses in nurse healthcare centers or primary care physicians and assessed the appropriateness of the information provided by these healthcare providers. The study protocol was approved by the Medical Ethics Committee of the University of Lausanne (ref. 166/02), and the study was performed in compliance with the Helsinki Declaration.

### Objectives

The primary objective of this study was to compare the quality of care provided to asylum seeking patients by different healthcare providers in a gatekeeping system (Figure 1). Beyond the technical and interpersonal aspects of care [25], quality of care was defined by Maxwell [26] as access to services, relevance to need (for the whole community), effectiveness (for individual patients), equity (fairness), social acceptability, efficiency, and economy. Our study focused on access to services and relevance to medical needs. We formulated 3 major questions: 1) Are the procedures used to identify the medical needs of asylum seekers similarly adequate between nurse practitioners and physicians? 2) Do asylum seekers have the same opportunity to access medical treatment when consulting a nurse practitioner and a physician? 3) Is the frequency of unnecessary medical procedures similar for nurse practitioners and physicians?

Secondary objectives were to describe the appropriateness of medical care for each separate setting within the gatekeeping system (Figure 1), to describe in detail each of the items used to assess appropriateness, and to evaluate the presence of serious events that could be the result of inappropriate care.

### Settings

We focused our assessment on four different structures (Figure 1) within the healthcare system, which have existed in Western Switzerland since 1996. The first is the Nursing Healthcare Centres (NHC) for asylum seekers, which are situated in four strategic areas of Western Switzerland (State of Vaud): 1 in a rural setting at the border of the country (Vallorbe) and 3 others in urban settings (Yverdon, Clarens, and Lausanne). The second is 6 general practices (GPs) of physicians who belong to a specific healthcare network for asylum seekers. All are located in the most important urban setting in terms of the number of asylum seekers (Lausanne). The third is an academic medical outpatient clinic (MOC) specializing in the care of vulnerable populations and a community health approach (Lausanne). The fourth is the emergency ward of the State University Hospital of Lausanne (UH). The major differences between these settings are: 1) the absence of nurse practitioners at the GPs, MOC, and UH; 2) the absence of physicians at the NHC; 3) more limited access to the GPs, MOC, and UH; 4) differences in the type of care at the UH versus the MOC (patients arriving at the university hospital will be sent to the UH for severe cases and to the MOC for nontraumatic ambulatory care); 5) supervision by a chief resident in the MOC, UH, and NHC but not at the GPs, which are managed by independent physicians; 6) better access to interpreters within the network of the institutions (the MOC and UH) versus the NHC or GPs; and 7) easier access to complementary evaluations or advice from specialists in the UH and MOC versus the GPs and NHC.

### Health providers

Nurse practitioners were qualified nurses from the Swiss Red Cross or academic institutions (University of Basel) who had additional specific training for independent clinical decision making in terms of diagnosis, therapeutics, and referral for asylum seekers. Those postgraduate courses are provided in different academic settings (Universities of Lausanne, Geneva, Basel, and Bern) as part-time training to experienced nurses who have worked in various fields such as tropical medicine, women and child care, public health, and non-governmental organizations (NGO). Even if nurses are thereafter independent in their work at the NHCs, they also work in close collaboration (role of gatekeepers) with primary care physicians in the network for asylum seekers and can request support from the chief residents of the MOC of Lausanne. Physicians working for the network also have specific training focusing on the health needs of asylum seekers.

### Patients

Medical records of asylum seekers were randomly selected from a list of all asylum seekers who had consulted each setting between June and November 2003 using a computer-generated list of random numbers (Epi info 6.04 d; CDC, Atlanta, USA). Providers were asked to submit a copy of the patient's record. Only patients over 16 years of age were included. Among the 1687 asylum seekers who had consulted during the study period, we selected the first eligible 200 patients from the NHC, the first 50 eligible patients from the GPs, the first 100 eligible patients from the MOC, and the first 100 eligible patients from the UH.

### Assessment of medical records

A panel of experts assessed the appropriateness of medical care provided in each encounter. Three physicians analyzed all 450 medical records. One nurse practitioner specialized in the medical care of asylum seekers joined the group for the assessment of the 200 records collected from the NHC. She was deemed competent enough to evaluate the appropriateness of care provided in the NHC by virtue of her specific training and experience with asylum seekers as a nurse practitioner. The expert panel of physicians included 1) one senior registrar from the MOC, an internist holding a masters of science degree in public health, specializing in the care of vulnerable populations; 2) one private general practitioner who had worked with asylum seekers since the creation of the network in 1996; and 3) one resident in his third year of general practice (sensitive to the problems of migration).

Panel reviewers were asked to perform a pretest assessment of 20 medical records in order to homogenize the assessment procedure. These 20 medical records were among the 450 records analyzed.

Evaluation of the appropriateness of care focused on medical aspects of care and not the comprehensive approach needed by healthcare providers to cope with the psychosocial problems and social demands frequently presented by asylum seekers. We used Lang's [27] definition of appropriateness to assess adherence to good clinical practice, evaluating: 1) capacity to identify medical needs, 2) access to medical treatment for those who need it, and 3) "non-access" to unnecessary medical procedures.

Medical records were photocopied and used as the source of data. A case report form was provided to each expert, who was asked to assess adherence to good clinical practice for reporting for the medical history, clinical examination, complementary investigations, referral, and treatment (Table 1). The experts were blinded to each other's scores. Blinding of healthcare settings was not possible since it was revealed in most medical records. Appropriateness of procedures for identifying medical needs was defined as the absence of inadequate components in the medical record (medical history, clinical examination, complementary investigation, and referral). The asylum-seekers' medical care needs were determined to have been met if the treatments were judged sufficient. The absence of unnecessary medical procedures was defined based on the criteria shown in Table 1. The predefined criteria for acceptance of assessment results were 75% concordance for the 4-person panel and 66% concordance for the 3-person panel. When these criteria were not achieved (e.g. when agreement could not be reached), an additional independent expert specializing in internal and tropical medicine was asked to make the final evaluation. The concordance between panel experts before attempting to reach concordance ranged from 0.62–0.81 for the NHC and 0.68–0.95 for the other settings.

**Table 1:** Evaluation chart for good clinical practice assessment

**Item**	**Adequate**	**Inadequate**	
**Medical History**	Major information needed to differentiate possible important diagnosis were reported	Insufficient	Important information for clinical decisions was not reported
		Excessive	Useless information collected which could either confuse the patient or the practitioner was reported
**Clinical Examination**	Appropriate examinations were used in light of the medical history which brings to a reasonable clinical decision were reported	Insufficient	Clinical examination which could have helped for clinical decisions was not reported
		Excessive	An unnecessary clinical exam was reported which could either confuse the patient or the practitioner
**Complementary investigations**	Investigations were done appropriately in light of the results of medical history and clinical examination and seemed essential for clinical decision.	Insufficient	Results of accessible complementary examination which could have been useful was not reported
		Excessive	Clinically unjustified laboratory tests were done
**Referral**	Decision to refer or not to refer was taken appropriately	Insufficient	Patient should be referred for additional care but wasn't
		Excessive	Patient was referred without it been necessary
**Treatment**	Adequate treatment was proposed having taken into consideration the diagnosis and eventual counter-indications.	Insufficient	Appropriate medical treatment not reported
		Excessive	Inappropriate treatment or incompatible treatment with clinical information was reported

Age, sex, country of origin, residence in the host country, and the main medical reasons for the consultation were also recorded. For each record the last visit documented was assessed. We also determined whether the patient was seen by a nurse only or had been referred to a general practitioner, a specialist, or the hospital (emergency ward). We also calculated the frequency of referrals by the general practitioners, the physicians of the MOC, and the staff of the emergency ward.

### Statistical analyses

**Figure 2:**
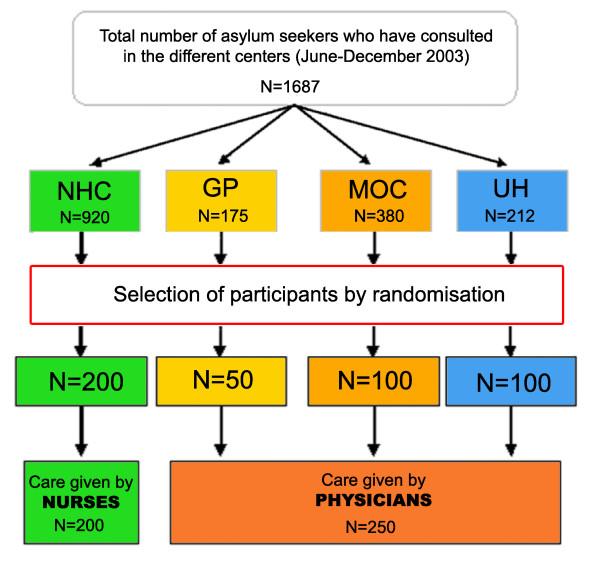
**Selection of participants**. NHC: Nurse Healthcare Centre. GP: General Practitioners. MOC: Medical Outpatient Clinic. UH: University Hospital emergency ward department.

Sample size was calculated assuming physicians' clinical decisions were appropriate in 95% of the consultations and based on our aim to detect an absolute difference of 10% between nurse practitioners and physicians with a power of 90% and a significance level of 0.05. Both groups were expected to include 209 patients. For practical reasons, this was rounded to 450, with 200 records from the NHC, 100 from the UH, 100 from the MOC, and 50 from GPs (Figure 2).

Patient data were assigned to two groups for analysis: one for patients whom had been seen by nurse practitioners, and one for patients whom had been seen by physicians. The absolute difference between groups was calculated for each parameter as well as for the three parameters combined, with a 95% CI. Group differences were analyzed with the χ^2 ^test, with significance set at p ≤ 0.05. All statistical analyses were performed using Stata 9.2 (College Station, Texas, USA)

## Results

### Description of asylum seekers

The mean age of asylum seekers was 32 years, ranging from a mean of 31 to 34 between the settings. Sixty percent of the asylum seekers were men. Patients from Sub-Saharan Africa (44%) were most highly represented and were mainly from Somalia (9.8%), Congo (6.2%), Angola (4.4%), Guinea (4.4%), and Sierra Leone (4.2%). The second most highly represented region of origin was the Balkans (33%), with 18.1% from Serbia, Kosovo, and Montenegro, and 13.1% from Bosnia. Patients from the Middle East (10%) primarily originated from Turkey (2.9%), Iraq (2.4%), Afghanistan (1.3%), and Palestine (0.9%). Other notable countries of origin were Algeria (2.2%), Sri Lanka (2%), Russia (1.8%), Georgia (1.8%), and Romania (1.3%).

Generally, patient characteristics did not differ greatly across the 4 healthcare structures; exceptions were the reason that led them to consult and the type of habitation (Table 2). Asylum seekers with gastrointestinal disorders or those that had an accident were more likely to consult the emergency unit (UH), whereas those with ear-nose-throat complaints were more likely to see the nurse practitioner (NHC). A higher percentage of asylum seekers who consulted a private practitioner (GP) (88%) lived in an apartment compared to the other settings (60–69%).

**Table 2:** Baseline characteristics according to site of encounter

	**NHC**	**GP**	**MOC**	**UH**	**Total**	
	n = 200	n = 50	n = 100	n = 100	n = 450	P values*
**Number of women [%]**	73 [36.5]	27 [54]	46 [46]	36 [36]	182 [40.4]	p = 0.066

**Mean age (SD)**	31.1(11.8)	34.2(12.6)	33(13.4)	31.7(13.1)	32 (12.6)	p = 0.364

**Origin [%]**						p = 0.106
Sub-Saharan Africa	99 [49.5]	25 [50]	43 [43]	33 [33]	200 [44.4]	
Balkan	65 [32]	13 [26]	37 [37]	35 [35]	150 [33.3]	
Middle/Far-East	20 [10]	5 [10]	7 [7]	13 [13]	45 [10]	
Others	16 [8]	7 [14]	13 [13]	19 [19]	55 [12.2]	

Habitation						p = 0.003
Apartment [%]	136 [68]	44 [88]	60 [60]	68 [68]	308 [68.4]	
Centre for refugees [%]	42 [21]	2 [4]	34 [34]	25 [25]	103 [22.9]	
Bomb shelter [%]†	22 [11]	4 [8]	6 [6]	7 [7]	39 [8.7]	

Main Health Problem						p < 0.001
Gastro-intestinal [%]	12 [6]	8 [16]	19 [19]	24 [24]	63 [14]	
Osteo-articular [%]	33 [16.5]	9 [18]	9 [9]	6 [6]	58 [12.9]	
Neurological [%]	15 [7.5]	7 [14]	12 [12]	15 [15]	49 [10.9]	
Dermatological [%]	20 [10]	4 [8]	12 [12]	1 [1]	45 [10]	
Ear-Nose-Throat [%]	38 [19]	4 [8]	8 [8]	1 [1]	40 [8.9]	
Accident [%]	7 [3.5]	2 [4]	6 [6]	27 [27]	41 [9.1]	
Other [%]	75 [37.5]	16 [32]	34 [34]	26 [26]	151 [33.6]	

* P-values were calculated with X^2 ^test for all variables except age were ANOVA was used.

† bomb shelter: as there was not enough space for asylum seekers in the centres created for them, the local government has decided to adapt the Swiss military bomb shelters as temporary living places for asylum seekers.

NHC: Nurse Healthcare Center, PCP: Primary Care Physician (GP+MOC+UH), GP: General Practitioners, MOC: Medical Outpatient Clinic, UH: University hospital (emergency ward department)

### Descriptive analysis of care

Of the 450 medical records that were analyzed, 4 had missing pages, making it impossible to assess the adequacy of the clinical examination (1 from GP and 2 from NHC) or the complementary investigations (1 from NHC).

For all settings, the least appropriate aspect of care was definition of patient needs through rigorous medical procedures. Use of inappropriate procedures to define medical needs was identified in 61 of 450 medical records (13.6%). By setting, the frequencies were 21% for the NHC, 12% for the GPs, 10% for the UH, and 3% for the MOC. Generally, the error was failure to report appropriate information from the clinical examination (40/61) or medical history (35/61 reports). For 21 records, both the history and the exam were inappropriate; 16 of these records were issued from the NHC. However, use of unnecessary procedures was identified in only 3 cases (0.7%).

A large percentage of the asylum seekers, 442/450 (97.8%), received the treatment they needed (Table 3). No significant differences were observed between healthcare settings. Among the remaining 8 patients, 2 from the NHC were not given the treatment they needed before seeing the referred physician a few days later; 6 others were seen by physicians who did not report any treatment in the medical record.

**Table 3:** Appropriateness of medical care

	GP (n = 50)	MOC (n = 100)	UH (n = 100)	**PCP **(n = 250)	**NHC **(n = 200)	Absolute Difference	
	n	n	n	n [%]	n [% ]	% [CI95%]	P-Value
**Defining needs**	44	97	90	231 [92.4]	158 [79]	13.4% [6.9;19.9]	p < 0.0001
Medical history	45	99	97	241 [96.4]	174 [87]	9.4% [4.2;14.6]	p = 0.0002
Clinical examination	47	98	95	240 [96]	170 [85]	11% [5.5;16.5]	p < 0.0001
Complementary investigations	50	99	95	244 [97.6]	199 [99.5]	-1.9% [-4;0.2]	p = 0.1056
Referral	50	100	96	246 [98.4]	197 [98.5]	-0.1% [-2.3;2.2]	p = 0.9321
**Access to treatment**	48	100	96	244 [97.6]	198 [99]	-1.4% [-3.7;0.9]	p = 0.2641
**Excessive medical care**	0	1	1	2 [0.8]	3 [1.5]	-0.7% [-2.7;1.3]	p = 0.4815

Overall appropriateness	42	97	90	229 [91.6]	157 [78.5]	13.1% [6.4;19.8]	p = 0.0001

GP: General Practitioners, MOC: Medical Outpatient Clinic, UH: University Hospital (emergency ward department), PCP: Primary Care Physician (GP+MOC+UH), NHC: Nurse Healthcare Centre.

Five patients received excessive care (1.1%). One patient who had not been seen by a healthcare provider was prescribed drugs over the phone (NHC). One patient was given a benzodiazepine without needing it (NHC). Two patients underwent unnecessary complementary evaluations (MOC and UH). One patient underwent excessive clinical examinations (NHC). None of the medical reports mentioned any serious complications that could have been related to inappropriate care.

At the NHC, 64 of 200 patients (32%) were referred to a general practitioner and 34 to a specialist (17%: 5% to a gynecologist, 3% to a dentist, 2% to an ophthalmologist, 2% to a surgical emergency ward, 2% to a medical emergency ward, and 3% to a psychiatric emergency ward). For physicians, 32.4% of the patients were referred to a specialist; referral rates were different across settings (18% for GP, 23% for MOC and 49% for UH).

### Difference between nurse practitioners and physicians

The null hypothesis that the quality of procedures used to identify the medical needs of asylum seekers was similar between nurse practitioners and physicians was rejected. Processes for defining medical needs were deemed appropriate for 92.4% of the reports from physicians and only 79% of the reports from nurse practitioners, a significant difference of 13.4% (p < 0.0001, 95% CI: 6.9–19.9].

In contrast, access to treatments was similar between groups (p = 0.264); 97.6% of patients consulting a physician and 99% of patients visiting the NHC obtained the treatment they needed.

Excessive care was the least frequent reason for inappropriate medical care (5/450 reports). Three reports (1.5%) from the NHC and 2 from physicians (0.8%) were deemed indicative of excessive care. The absolute difference of 0.7% (95% CI: -1.3–2.7) in favor of physicians was not significant (p = 0.481).

The overall appropriateness of medical care, applying Lang's definition [26], was 85.8% for the 450 participants, 91.6% for physicians, and 78.5% for nurse practitioners. The difference in overall quality between providers (13.1%, 95% CI: 6.4–19.8) was due to inappropriateness in the use of medical history and clinical examination.

## Discussion

Our results show that the capacity to define the medical needs of asylum seekers differs between nurse practitioners and physicians. In particular, the medical history and clinical examination were less well reported in NHC records. Nevertheless, access to treatment was achieved for more than 97% of asylum seekers (97.6% for primary care physicians and 99% for nurse practitioners), and there was not an excess of complementary evaluations or treatment. Thus, it appears that asylum seekers are appropriately cared for through adapted means.

### Defining needs

Procedures for medical history and clinical examination were rated less appropriate than those for complementary investigations and referral. It is widely recognized that recording an accurate medical history for asylum seekers is difficult because of language barriers. Thus, the lesser appropriateness of these procedures for the NHC may be due to the unavailability of full-time translators at that facility, whereas such services were more available in the MOC and the UH. Indeed, access to translators and cultural mediators is important and should be considered a key aspect of the appropriateness of the care given to patients with limited language proficiency [28,29].

When considering the clinical examination, it is not surprising that nurses scored lower in appropriateness, since their training is focused more on sociomedical aspects than on procedures for examining patients. Primary care for asylum seekers could therefore be improved by specifically training nurses to perform simple clinical examinations.

Similarly, the relative weakness in acquisition of medical history in the general practice settings might also relate to poor communication between patient and caregiver. Medical care was the most appropriate at the medical outpatient clinic. This was expected, since physicians in this department are sensitive to the problems of vulnerable populations and receive training to identify their specific problems.

### Access to treatment

Access to treatment was evaluated to be appropriate and similar between nurse practitioners and physicians. Indeed, treatment and referrals were judged appropriate in more than 97% of cases, across all 4 settings. This finding is consistent with the results of randomized clinical trials comparing the effectiveness of care between primary care physicians and nurse practitioners [21,22]. Therefore, it appears that the gatekeeping system can provide patients with the treatment they need, although the processes to define these needs are not always appropriate. There are multiple possible explanations for this apparent paradox. Nurse practitioners are experienced and receive postgraduate training in primary care. They are trained to identify signs and situations where they should consider seeking the advice of the MOC's chief residents or referring the patient to another healthcare provider (gatekeeping). They are apparently able to clearly identify cases requiring further competency and refer these patients appropriately (32% were referred to a GP and 17% to specialists). Thus, the gatekeeping process allows asylum seekers to see the specialist they need. This is reflected in the high rate of referral to a specialist (32.4%) when asylum seekers consult a physician, as compared to the Swiss patient population (5.1%) [30]. This clearly demonstrates the gatekeeping role played by nurses, which reduces the number of consultations by general practitioners and steers patients toward the adapted care setting within the network.

### Excessive care

Surprisingly, nurse practitioners did not use an excess of complementary evaluations for any of the 250 patients. In other settings, nurses are usually more likely to do so [19,20]. There are multiple possible reasons for this difference. First, Red Cross nurses are trained to treat patients with minimal means. Second, supervision by chief residents may influence the decision against complementary evaluations. Third, patients requiring many complementary evaluations are more likely to be referred to physicians, because of the complexity of the case. Fourth, NHCs are not equipped to perform many complementary evaluations. Thus, the gatekeeping system seems to be an appropriate means to encourage healthcare providers to limit complementary evaluations to necessary circumstances.

Furthermore, asylum seekers seldom received inappropriate or incompatible treatments when visiting physicians (0.8%) or the NHC (1.5%). Again, this could be due to the existence of a network, which gives nurse practitioners the opportunity to ask for advice or refer patients to other competent health providers when needed.

### Overall appropriateness

The global quality assessment indicated that the percentage of encounters that were appropriate with regard to all examined items was 78.5% for NHC and 91.6% for primary care physicians. Although these numbers might be considered suboptimal, we underscore that our assessment was very stringent-all items had to be appropriate for a satisfactory overall assessment. Moreover, if we examine the appropriateness of healthcare in patients from U.S. communities, we find that only 54.9% of patients receive recommended care [31]. Furthermore, none of the 450 patients experienced any serious complication due to inappropriate care. Finally, physicians and nurses are not trained to identify the same needs. Our study only evaluated the appropriateness of answering the needs usually identified by physicians but did not consider the psychosocial needs which nurses are often more trained to answer to. Thus, we believe that this study provides evidence for reasonable quality of medical care for asylum seekers at various healthcare sites. Thus, this healthcare network constitutes an interesting model and deserves the attention of the national and international authorities who are responsible for the medical care of asylum seekers. Economic evaluation of this kind of network [32,33] as well as qualitative assessment of patients (satisfaction with regard to the care received) and healthcare providers (satisfaction with regard to the care provided) [34] will complement our evaluation of the appropriateness of medical care.

### Limitations

Our study has several limitations, the major one being selection bias due to the fact that the patients visiting the different settings were dissimilar. However, these differences were inherent to the network itself (i.e. more critical care in the emergency ward of the UH). Also, our study did not assess the actual encounters, but instead the information available in the medical records. The panel's judgments about the appropriateness of examination, investigation, treatment, and referral were reliant on what was recorded by the clinician. It is likely that some of the information gathered during the consultation was not transcribed in the patient file, and thus unavailable to the panel. Also, while interpersonal relations are crucial for appropriate medical care within such a network, this component was not investigated. Also, the presence of a senior registrar of the MOC on the expert panel could have created a group bias among the evaluators. Finally, it is important to point out that our evaluation of the quality of this gatekeeping system managed by nurse practitioners focused on the appropriateness of medical aspects of care and did not integrate the comprehensive approach nurses might use to resolve the range of psychosocial problems and social demands frequently presented by asylum seekers.

## Conclusion

This is the first study to evaluate the appropriateness of the procedures nurse practitioners use to make clinical decisions regarding asylum seekers. Involving nurse practitioners in a network healthcare system for asylum seekers seems to be useful in guaranteeing access to treatment for this vulnerable population. The close collaboration between nurses and physicians could compensate for nurses' weaknesses in recording histories and conducting clinical examinations. We therefore consider a gatekeeping system managed by nurse practitioners to be an appropriate healthcare model for asylum seekers, provided that a strong medical network exists that allows referral to the most appropriate medical competence, when needed. The main lesson that we might consider is that nurse practitioners must continue to undergo specific training that includes sensitization to the health problems of these vulnerable populations and a focus on recording medical history and performing targeted clinical examinations.

## Competing interests

The author(s) declare that they have no competing interests.

## Authors' contributions

PB carried out the design of the study and it's conception, participated in the acquisition, analysis and interpretation of data, and drafted the manuscript. FA Participated in the design of the study, performed the acquisition of the data, participated in the analysis and interpretation of data and helped to draft the manuscript. BB participated in the design of the study and in the analysis and interpretation of data. PV performed the statistical analysis and drafted the manuscript. AP participated in the conception and design of the study, in its coordination and helped draft the manuscript. BG participated in the acquisition, analyses and interpretation of data and drafted the manuscript. All authors read and approved the final manuscript.

## Pre-publication history

The pre-publication history for this paper can be accessed here:


